# Minimally access via left anterior mini-thoracotomy for repair of adult subarterial ventricular septal defects

**DOI:** 10.1186/s13019-017-0611-7

**Published:** 2017-06-12

**Authors:** YunFei Liao, Xiang Long, ShuQiang Zhu, Jun Tu, Hua Wen, JianJun Xu, YongBing Wu

**Affiliations:** Department of Cardiothoracic Surgery, The Second Affiliated Hospital of Nanchang University, Nanchang, Jiangxi Province 330006 People’s Republic of China

**Keywords:** Minimally invasive cardiac surgical techniques, Left anterior mini-thoracotomy, Subarterial VSDs, Adults

## Abstract

**Background:**

Minimally invasive cardiac surgical techniques are increasingly applied in the treatment and management of a variety of adult ventricular septal defects (VSDs). However, repair of adult subarterial VSDs via left anterior mini-thoracotomy is rarely reported. The present study aimed to determine the feasibility and safety of the left anterior mini-thoracotomy for the repair of adult subarterial VSDs.

**Methods:**

Twenty-seven adult patients underwent repair of subarterial VSDs via left anterior mini-thoracotomy. The approach includes two options for skin incision access, longitudinal and transverse skin incisions. The skin incision length was 4.1–6.1 cm (mean, 5.1 ± 0.6 cm). The closure of the VSDs was obtained through the main pulmonary artery under direct visualization.

**Results:**

Successful repair of the defects was achieved in all the patients. No patients died or converted to median sternotomy. Average durations of cardiopulmonary bypass (CPB) and aortic cross-clamp were 102.5 ± 13.6 min (range, 85–127 min) and 54.6 ± 6.9 min (range, 45–66 min), respectively. No patients required blood transfusion. The average postoperative hospital stay was 5.1 ± 0.7 days (range, 4–6 days). There were no postoperative complications related to the operative procedures or peripheral cannulation. During the follow-up of 5.4–32.3 months, no patients were found to have residual shunt, wound infections, pericardial effusion, neurologic or other complications.

**Conclusion:**

Our experiences demonstrate that minimally invasive cardiac surgical technique via left anterior mini-thoracotomy can be served as a novel, feasible and safe alternative for the repair of adult subarterial VSDs.

## Background

Conventional median sternotomy is the most common surgical access used for cardiac surgery; it is extensively applied in the repair of subarterial ventricular septal defects (VSDs). To date, intracardiac repair under direct visualization via median sternotomy is still considered the gold standard for treatment of VSDs [1]. However, the conventional surgery always accompanies by long midline or thoracotomy skin incisions, postoperative pain and poor cosmetic effects. Occasionally, mediastinitis and osteomyelitis may make the repair of VSD troublesome [2]. In the past few years, interventional occlusion and minimally invasive cardiac surgery have gained popularity in the treatment of VSDs [3, 4]. However, though interventional occlusion has been extensively applied in the treatment of perimembranous and muscular VSDs, some controversies exist in its application due to its complexity and its potential to damage the aortic valve; thus its application to some extent has been limited [3]. By contrast, minimally invasive cardiac surgery has been increasingly applied to adult coronary revascularization, valvular surgery and congenital heart disease, especially in recent decades [5]. Minimally invasive cardiac surgery includes two primary accesses, right mini-thoracotomy and lower partial sternotomy [4, 6]. A novel alternative, left anterior mini-thoracotomy, is less commonly used in the repair of adult subarterial VSDs and is less frequently reported in previous studies.

The present series aimed to determine the feasibility and safety of minimally invasive cardiac surgical technique via left anterior mini-thoracotomy for the repair of adult subarterial VSDs. From June 2013 to October 2016, we performed minimally invasive repair of subarterial VSDs for 27 adult patients via the left anterior mini-thoracotomy. By analyzing the clinical data of these 27 patients, such as CPB time, cross-clamp time, postoperative drainage volume, mechanical ventilation time, intensive care unit (ICU) stay and postoperative hospital stay, etc., we concluded that minimally invasive cardiac surgery via left anterior mini-thoracotomy could be served as a novel, safe and feasible alternative for the repair of subarterial VSDs.

## Methods

### Inclusion and exclusion criteria

This approach described herin is mainly applicable to the adult single subarterial VSDs patients without any other intracardiac lesions. The body weight of these patients should be controled in 35–80 kg (according to our exprimences). Some difficults exist in constructing an extracorporeal circulation for those too light patients (<35 kg), while for those who are too heavy (>80 kg), it has some trouble to expose the operative field for the thick cortex. In addition, patients who accompanied by moderate or severe aortic insufficiency are not the suitable cohort, for these patients should receive an extra aortic vulve replacement. Patients who simultaneously suffer severe pericardial adhesions are also not the suitable cohort, for it is difficult to expose the operative field.

### Patients

Twenty-seven adult patients (12 male, 15 female) with subarterial VSDs were selected to undergo repair of subarterial VSDs by this minimally invasive cardiac surgical technique via left anterior mini-thoracotomy. Among these patients, the average age and body weight were 28.3 ± 9.7 years (range, 19–46 years) and 55.4 ± 10.6 kg (range, 40–73 kg), respectively. By preoperative examinations, such as transthoracic color doppler ultrasound or transesophageal echocardiography (TEE), all patients were confirmed to have isolated subarterial VSD and no other intracardiac malformations. The average VSD size was 7.9 ± 4.4 mm (range, 4.0–18.0 mm). According to New York Heart Association (NYHA) classifications, 16 were classified as grade I and others were grade II. The average ejection fraction was 58.5 ± 7.2% (range, 50–72%). 7 patients were found with aortic valve regurgitation (4 trivial / 3 mild). Mild pulmonary artery hypertension was found in 2 patients, pressures were 31 and 39 mmHg, respectively. No other anomalies were found in all patients.

The operative policy to use minimally invasive cardiac surgical technique for the repair of subarterial VSDs was approved by the appropriate hospital authorities. Written consents were obtained from the patients and the family members before operation.

### Methods

The patient was placed in the supine position with the right groin exposed and the arms naturally and respectively situated on the sides of the body. The defibrillator electrodes were routinely adhered to the body surface preparing for cardioversion when necessary after VSD being repaired. After single lumen endotracheal intubation and the induction of general anesthesia, transesophageal echocardiographic monitoring was set up and the diagnosis of subarterial VSD was further confirmed. Then, the right inguinal fossa was obliquely incised to anatomize the femoral vessels, and then 17–21 F (Medtronic, USA) and 22–28 F (Edwards, USA) cannulas were inserted into the femoral artery and vein, respectively, to establish cardiopulmonary bypass (CPB). Venous cannula was delivered to the right atrium under TEE guidance, with the tip located in the superior vena cava. Systemic hypothermia was begun immediately after the initiation of CPB.

**Fig. 1:**
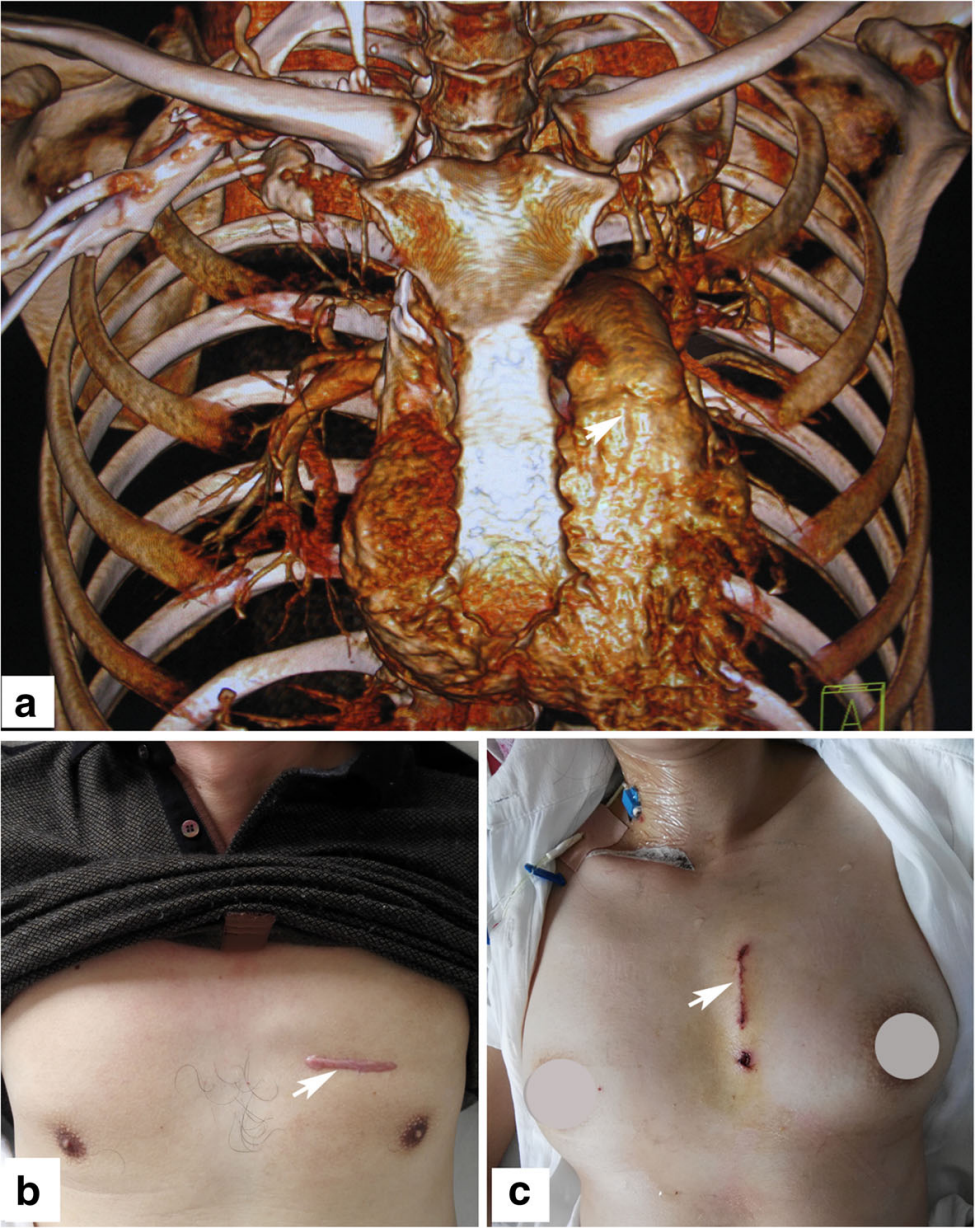
Minimally invasive cardiac surgical technique via left anterior mini-thoracotomy is used for the repair of adult subarterial VSDs. **a** The chest CT scan combined with 3-dimensional reconstruction is used for positioning the subarterial VSD site (the *white arrowhead* shows). The *arrowheads* indicate the skin incision accesses used for repair of subarterial VSDs via left anterior mini-thoracotomy, **b** transverse incision was only chosen by male patients, **c** longitudinal incision was mainly chosen by female patients

According to the positioning results of the chest computed tomography (CT) scan combined with 3-dimensional reconstruction (Fig. 1a), the most optimal skin incision access was defined as the intercostal space nearest to the VSD site. According to our experiences, the incision access was most commonly located in the second or third intercostal space. In the present series, access was via the second intercostal space in 7 patients and the third intercostal space in 5 patients. Generally, a transverse incision access across the intercostals space in the left parasternal region was chosen only for male patients (Fig. 1b). In this type of incision, no matter which intercostal space was chosen as the incision access, the sternal extremity of the third costal cartilage was divided, without resection, to increase exposure. The pleura were then incised to open the pleural cavity and expose the heart.

For female patients, in order to avoid injury to mammary tissues, the above incision access was not appropriate. Thus, a longitudinal incision (approximately the same length as the transverse incision), as a second minimal incision access in this approach, roughly adjoin to the left margin of sternum, was used when operating on female patients (Fig. 1c). The longitudinal incision ranges from the lower margin of the second rib to the upper margin of the fourth rib. The sternal extremity of the third costal cartilage was divided, fractured, and inverted interiorly to increase exposure. There was no need to open the pleural cavity with this type of incision access.

**Fig. 2:**
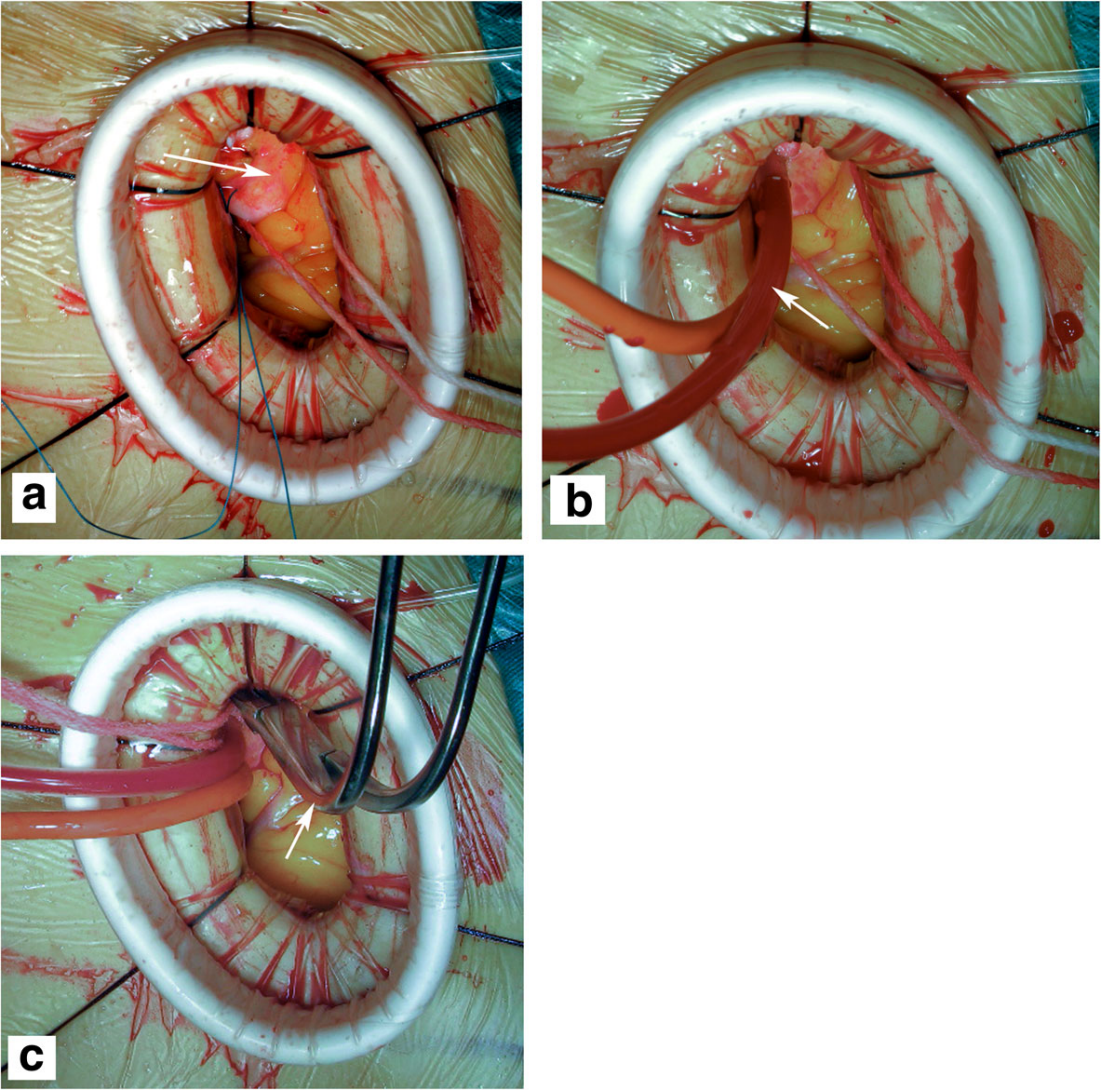
The aortic cross-clamping in this approach is technically feasible. **a** The *arrowhead* indicates the fully exposed root of the aorta, where cannulation and aortic cross-clamping would be happened. The *arrowheads* indicate the antegrade cardioplegia cannula (**b**) and the blocking clamp (**c**), respectively

**Fig. 3:**
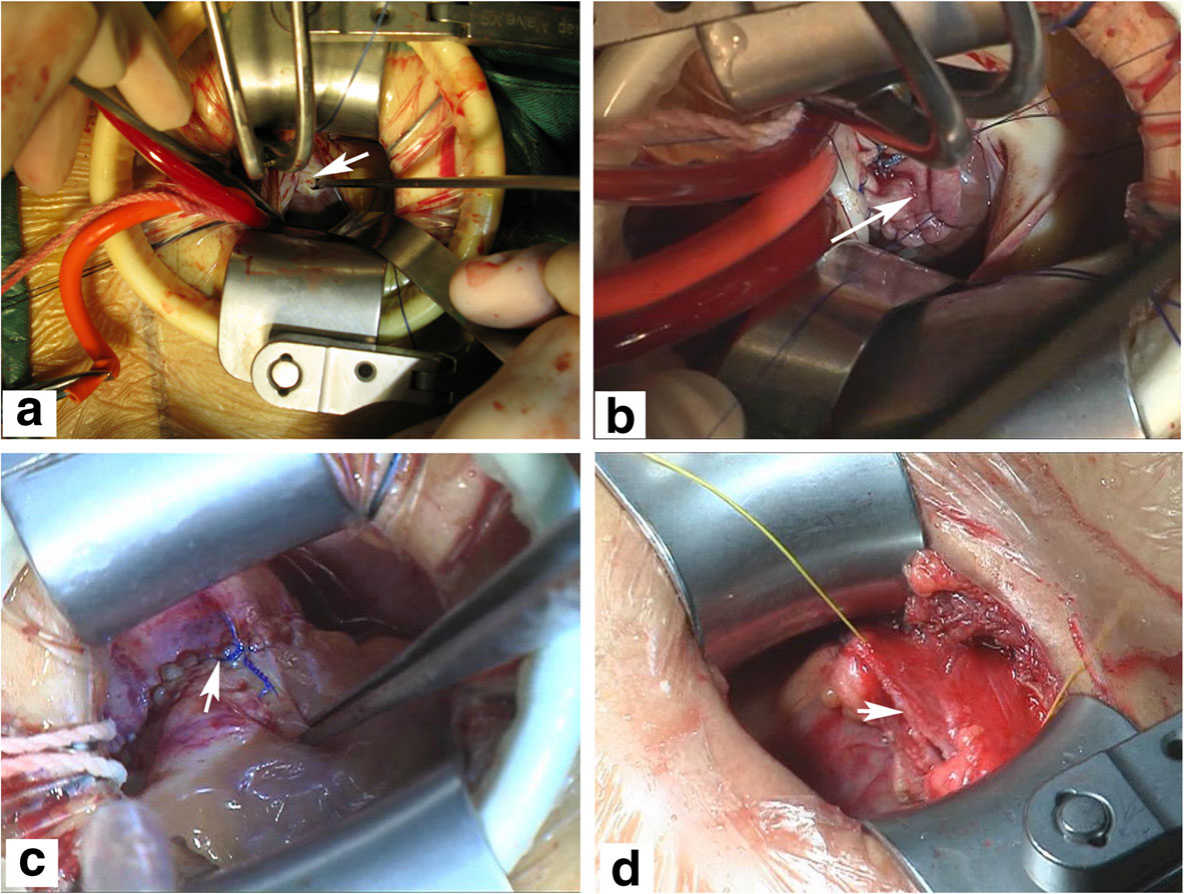
Pictures show the closure of subarterial VSD under direct visualization. **a** After the transpulmonary arteriotomy having been performed, the VSD site was exposed (the *arrowhead* shows) under direct visualization. **b** The *arrowhead* indicates that the subarterial VSD is closing with a bovine pericardial patch. **c** After the VSD being repaired, pulmonary artery was closed with 5–0 prolene running sutures (the *arrowhead* shows). **d** Sequencially, the pericardium was closed loosely with running sutures (the *arrowhead* shows)

A soft tissue protector and chest wall retractor were routinely used to widen the incision and expose the operative field. Subsequently, the pericardium was longitudinally incised and suspended to expose the roots of the aorta and pulmonary artery (Fig. 2a). Then, the CO_2_ insufflation tube was inserted into the pericardial cavity across the soft tissue protector, and CO_2_ was continuously infused into the operative field. Under CPB, the tissues between the aorta and the pulmonary artery were anatomized and the aortic traction belt was used to facilitate blocking of the ascending aorta. The antegrade cardioplegia cannula was inserted to the root of aorta (Fig. 2b). The antegrade cold blood cardioplegia solution was infused into the coronary orifice after aortic cross-clamping (Fig. 2c). Ice was routinely put onto the surface of the heart to protect the myocardium. After cardiac arrest, the transpulmonary arteriotomy was performed to expose the VSD (Fig. 3a), blood within the left ventricular system was continuously suctioned out through the VSD by a small soft tube connected to the cardiotomy suction system to maintain a clean operative field. Subsequently, the VSD was repaired using a bovine or autologous pericardial patch with running sutures under direct visualization (Fig. 3b).

Before complete closure of the VSD, the air in the left ventricle was evacuated by rotating the operative table in all directions. After resuscitation of cardiac arrest, the VSD was again examined to exclude possible residual defect. The electrocardiogram showed that sinus rhythm was recovered in all patients. Then, the pulmonary artery was closed with running sutures (Fig. 3c), and CPB was gradually withdrawn after rewarming of the patient. Sequentially, the pericardium was closed loosely with running sutures (Fig. 3d). The femoral arteriotomy and venotomy were closed with 5–0 and 6–0 prolene interrupted sutures. Hemostasis and closure of the incisions were performed smoothly.

## Results

The VSDs were repaired successfully in all patients with the use of bovine or autologous pericardial patches. No patients died or converted to median sternotomy. The average incision length was 5.1 ± 0.6 cm (range, 4.1–6.1 cm) (Fig. 1b and c). The cosmetic result was very satisfactory. The average CPB and aortic cross-clamp time was 102.5 ± 13.6 min (range, 85–127 min) and 54.6 ± 6.9 min (range, 45–66 min), respectively. No patients required blood transfusion. The mean mechanical ventilation time was 6.9 ± 1.1 h (range, 4.9–8.5 h). Average intensive care unit (ICU) stay was 17.7 ± 1.7 h (range, 15–20 h). Postoperative drainage volume within the first 24 h ranged from 20 to 102 ml (average, 65.0 ± 30.5 ml). Postoperative hospital stay ranged from 4 to 6 days (average, 5.1 ± 0. 7 days).

The average duration of follow-up was 15.4 ± 2.3 months (range, 5.4–32.3 months) after hospital discharge. During the periods of follow-up, no patients were found to have any residual shunt by transthoracic echocardiography. There were no wound infections, pericardial effusion, neurologic or other late complications.

## Discussion

Subarterial ventricular septal defects (VSDs) are located in the right ventricular outflow tract, which is in close proximity to the pulmonary artery valve. Usually, the upper margin of the defect lies in the fibrous ring between the pulmonary and aortic valves, and the lower margin extends to the supraventricular crest. Anatomically, subarterial VSDs are defined as conal septal defects [7]. They represent 5–7% of all VSDs [7, 8].

In this series, 27 adult patients were operated on to repair the subarterial VSDs with the use of minimally invasive cardiac surgical technique via left anterior mini-thoracotomy. The VSDs were all closed under direct visualization through a minimal skin access approximating to 4–6 cm. All the patients recovered rapidly with decreased surgical traumas and satisfactory cosmetic results. All the patients were discharged safely, with no mortality, no morbidity and no obvious postoperative complications.

Conventionally, intracardiac repair under direct visualization through median sternotomy has long been considered safe and effective treatment for subarterial VSDs, but this approach produces significant surgical traumas and leaves a long midline scarring [9]. For most adult VSD patients, the major concern is not the mortality or morbidity associated with repair surgery, but rather cosmetic problems arising from surgical scarring as a result of median sternotomy [10]. Researchers have explored transcatheter interventional occlusion and periventricular occlusion to determine whether these approaches can be feasibly applied in the treatment of subarterial VSDs [3, 11]. However, due to low success rate and increased incidence of postoperative complications, including residual shunt, arrhythmias, hemolysis, thromboemboli, aortic valve regurgitation, and translocation of closure device, the short- and long-term outcomes of these approaches are unfavorable [11–18]. Liu et al. [14] reported intraoperative device closure of subarterial VSDs in 62 cases. Overall, 16.1% of these cases were converted to full median sternotomy due to tricuspid regurgitation, aortic valve regurgitation, or residual shunt, and the overall success rate was only 83.9%. Additionally, the incidence of early- and late-stage complications of this approach was up to 19.2 and 3.8%, respectively [14].

To increase success rates, improve cosmetic results, and reduce surgical traumas, minimally invasive cardiac surgery has been increasingly applied in the treatment of VSDs, and the number of relevant literatures is increasing [1, 5]. Minimally invasive cardiac surgical techniques usually include the right anterior lateral minimal incision, right axillary minimal incision, and lower sternal minimal incision [6, 19, 20]. In addition, VSDs can also be repaired by robot via a minimally invasive access [21]. However, minimally invasive repair of subarterial VSDs via left anterior mini-thoracotomy is rarely reported. The present series adopted minimally invasive access via left anterior mini-thoracotomy for repair of adult subarterial VSDs.

Meticulous preoperative examination for the accurate diagnosis of VSD type is mandatory for successful outcomes. The minimally invasive access via left arterial mini-thoracotomy is specially designed for simple adult subarterial VSD--the minimal access is too small to simultaneously repair other intracardiac anomalies, it is important to confirm the diagnosis of subarterial VSD and exclude other intracardiac malformations by preoperative examination, such as transthoracic echocardiography or transesophageal echocardiography (TEE). In addition, accurately choosing the skin incision access is an extremely critical step for this approach. Thus, accurate preoperative positioning of the VSD is of great significance. We adopted chest computed tomography (CT) scan combined with 3-dimensional reconstruction for positioning the VSD site. According to the positioning result of preoperative chest CT, the intercostal space nearest to the VSD was considered as the most optimal skin incision access, across where aortic cross-clamping and exposing the VSD could be simultaneously achieved. After positioning the intercostal space, we should incise the skin along the upper edge of the lower rib to avoid the intercostal vessels to decrease hemorrhage. Anatomically, regardless of the intercostal space chosen as the incision access, the sternal extremity of the third costal cartilage must be divided, without resection, to increase exposure. It should be noted that, after the heart was arrested, the transpulmonary arteriotomy should be performed and blood within left ventricular system should be suctioned out through the VSD with the use of a small soft tube to keep the operative field clean and reduce the resistance of moving the heart, making it easier to pull the VSD site up to the center of the incision, which allowed us to repair the VSD under direct visualization.

Lin et al. [2] reported a video-assisted endoscopic technique under femoro-femoral cardiopulmonary bypass for the repair of subarterial VSDs in 11 patients using a transverse incision in the third or fourth intercostal space of the left parasternal region. In his procedures, the ascending aorta was not clamped, and the operation was performed under hypothermic fibrillatory arrest. However, this approach requires special surgical instruments and a long-term learning curve, which is not conducive for promotion. Additionally, operation under hypothermic fibrillatory arrest can’t provide optimal myocardial protection [10]. In order to modify this approach, we recommend minimally invasive access via left anterior mini-thoracotomy for simultaneously clamping the ascending aorta and closing the VSD under direct visualization. According to our experiences, minimally invasive access via left anterior mini-thoracotomy could provide adequate exposure of the VSD and sufficient myocardial protection. Moreover, it doesn’t require special instruments and have no much difficulty in terms of operation.

For female patients, Jung et al. [10] advocated the anterolateral mini-thoracotomy of the breast as the incision access. Roughly starting from the sixth rib, the mammary tissues were dissected through the thoraco-fascia plane to expose the third rib. Although the incision access is hidden, it can potentially damage the mammary tissues and thus is more likely to accelerate fat liquefaction [10]. In order to avoid injury to the mammary tissues and retain cosmetic results, we chosen a longitudinal minimal incision access in the left parasternal region for female patients who present with subarterial VSDs. Most of the steps are the same as the transverse incision except for the process of the third rib cartilage. For the longitudinal incision, the sternal extremity of the third costal cartilage should be divided, fractured, and inverted interiorly to increase exposure, while the third costal cartilage only needs to be divided for the transverse incision. So, for the longitudinal incision, the third costal cartilage should be reconstructed after the VSD being closed.

The prominent advantage of minimally invasive cardiac surgical techniques is the avoidance of sternotomy [2]. The minimally invasive technique can reduce the injury to patients and postoperative complications to a minimum [2]. In this present series, we analyzed the clinical data of these 27 patients who underwent repair of subarterial VSDs via left anterior mini-thoracotomy. According to analytic results, the approach brought very few surgical traumas and haemorrhage, all the patients can obtain a fast postoperative recovery. For most patients, they can discharge the hospital 5 days after the operation. Notably, the cosmetic effect was very gratifying, which was the uppermost superiority of this approach and as well as the prime objective of the patients.

## Limitations

However, the new approach has potential limitations. Subarterial ventricular septal defect (VSD) accounts for 5–7% of all the VSDs, mainly prevailing in older children and adolescents, adult subarterial VSD is relatively scarce. For older children and adolescents with light body weight and small body size, some difficulties exist in constructing peripheral cardiopulmonary bypass, so the novel technique presented in this series is not appropriate for this cohort; thus its scale of application is limited. But, with the accumulation of experiences and the development of associated techniques, the novel approach might break through age, body weight, body size and other potential restrictions, and could be extensively chosen by all the subarterial VSD patients and even by patients with other types of VSD, who require surgical intervention.

## Conclusion

The preliminary results adequately demonstrate that minimally invasive cardiac surgical technique via left anterior mini-thoracotomy can be served as a novel, safe and feasible alternative for the repair of subarterial VSDs.

## Abbreviations

CPB: Cardiopulmonary bypass
CT: Computed tomography
ICU: Intensive care unit
NYHA: New York Heart Association
TEE: Transesophageal echocardiography
VSD: Ventricular septal defect
